# The effect of increasing the gaps between the front teeth on torque and intrusion control of the incisors for anterior retraction with clear aligners: a prospective study

**DOI:** 10.1186/s12903-024-03867-w

**Published:** 2024-01-20

**Authors:** Ni Li, Xiao Lei, Yuan Cao, Lu Liu, Yanqi Zhang, Qiang Ning, Linyuan Gui, Fang Jin

**Affiliations:** State Key Laboratory of Oral & Maxillofacial Reconstruction and Regeneration, National Clinical Research Center for Oral Diseases, Shaanxi Clinical Research Center for Oral Diseases, Department of Orthodontics, School of Stomatology, Air Force Medical University, Xi’an, China

**Keywords:** Clear aligner, Extraction treatment, Anterior teeth retraction, Torque, Anchorage

## Abstract

**Objective:**

To investigate the effect of sequential distalization on increasing gaps in the maxillary anterior teeth, focusing on the control of torque and three-dimensional teeth movement during anterior retraction with clear aligners in extraction cases.

**Methods:**

We recruited 24 patients who were undergoing extraction bilateral maxillary first premolars with clear aligners. According to a predetermined increment in the spaces between the maxillary anterior teeth, the patients were divided into three groups: those with no gap (9 cases), a 0.5 mm gap (6 cases) and a 1.0 mm gap (9 cases). In each group, a 2.0 mm en-mass retraction was applied on the anterior teeth. Plaster casts of the upper full dentition were obtained both before and after a 2 mm retraction. The palatal folds were used to overlap each pair of models. The three-dimensional movement of the teeth and the change of torque for the anterior teeth were subsequently analyzed using Geomagic Studio 2014 software.

**Results:**

The change in torque in the groups with added gaps was significantly smaller than that in the group with no gaps (*P* < 0.05). There was no significant difference in this respect when comparing the group with a 0.5 mm gap added to the group with a 1.0 mm gap was added (*P* > 0.05). In the labial-lingual and vertical directions, the displacements of the central and lateral incisors were smaller in the groups with additional gaps compared to those in the groups without gaps (*P* < 0.05). However, there was no significant difference observed when comparing the group with a 0.5 mm added gap to the group with a 1.0 mm added gap (*P* > 0.05). Then, a comparison was made between the displacement of the second premolar to the second molar in the mesial-distal direction across all groups. The study revealed that the anchorage molars in the group without gaps demonstrated significantly smaller displacement compared to those in the group with additional gaps (*P* < 0.05).

**Conclusion:**

Advantages were observed in controlling the torque of the anterior teeth and achieving a desired pattern closer to normal bodily movement by sequentially distalizing the maxillary anterior teeth gaps. Increasing the gaps between the maxillary anterior teeth also resulted in improved control of the vertical direction of the anterior teeth. However, this retraction strategy necessitates substantial protection of the anchorage molars.

## Introduction

Clear aligners have clear advantages in terms of visibility, comfort, aesthetics, and reducing of chair-side operation time. Nevertheless, it is important to be considered. For example, the relatively poor ability to precisely control complex teeth movement; this represents a major drawback of clear aligners from a clinical perspective [1]. In the 1980s, clear aligners were deemed inappropriate for patients who were undergoing teeth extraction [2]. Due to advancements in biomechanics and the progress in materials science, the use of clear aligners in cases requiring teeth extraction cases is no longer considered a contraindication. However, it does create a challenge for clinicians. The primary considerations in cases involving tooth extraction relate to the retraction of the anterior teeth and closing the extraction gap. Inadequate control of torque and three-dimensional movement of anterior teeth by clear aligner appliances heightens the likelihood of teeth inclination, torque loss, lingual inclination, extrusion, and overbite deepening during the teeth retraction process [3]. This, in turn, results in a complex treatment plan that prolongs the duration of treatment.

Various techniques are employed to improve dental control in clinical practice. Firstly, attachments that closely fit the appliance to the crown can enhance the retention force and increase the contact area. Therefore, the application of accessories can improve the efficiency of treatment [4]. However, the shape and position of the attachments are artificially established, and the operation mechanisms and reliability of this method are still uncertain [5]. Furthermore, employing various retraction strategies, such as the stepwise retraction of canines and incisors, may lead to improved control of teeth; however, it could also result in a substantial extension of the treatment duration. Thirdly, over-correction may be pre-set to improve our ability to achieve efficient tooth movement. Nevertheless, there is a lack of established methods for determining an appropriate level of over-correction.

Clear aligners generate orthodontic forces through elastic deformation of the appliance, differing from the traditional fixed appliances. Additionally, the transmission of force depends on the proper wrapping around the crown. Therefore, it is crucial to focus on the wrapping and fitting of the crown with the appliance. In a previous study, Tepediion et al. introduced interproximal enamel reduction to create gaps between anterior teeth, and subsequently assessed the changes in torque in the anterior teeth of subjects following the use of 12 pairs of appliances. The authors reported that there was no statistically significant difference between the actual torque and the preset torque [6]. In clinical practice, he stepwise strategy of canines distalization and incisors retraction is frequently employed to close the extraction gap, thereby increasing the wrapping area and achieving effective control of the canines. Hence, we formulated a hypothesis that the expansion of the wrapping area of the appliance in the mesial-distal direction of the incisors could be enhanced by sequentially distalizing the anterior gaps. This enhancement may improve our ability to control teeth and prevent the “pendulum effect” or “roller coaster effect” during retraction. Therefore, a prospective clinical trial was conducted on extraction cases to examine the impact of retraction with or without increasing anterior teeth gaps on the control of anterior teeth torque and three-dimensional teeth movement in patients utilizing clear aligners.

## Materials and methods

### Participants

A total of 24 patients was recruited from the Orthodontics Department of Stomatology Hospital of The Air Force Military Medical University. The treatment plan involved extraction of bilateral maxillary first premolars, followed by clear aligner therapy according to clinical examination, cephalometric and model analyses. Out of the 24 patients recruits, there were 18 females and 6 males with a mean age of 28 ± 4.24 years. A total of 240 teeth were incorporated into this study.

Inclusion criteria for patients

1. Malocclusion involving the extraction of the bilateral maxillary first premolars has been planned
2. A $\underset{\_}{1}-$ NA angle within the normal range (22.8 ± 5.7°)
3. Crowding of the anterior teeth≤4 mm
4. Permanent dentition characterized by fully erupted second molars
5. A well-developed hard palate with no cleft palate and clear, complete palatal folds
6. No history of orthodontic treatment, no fixed prosthesis
7. Patients voluntarily accepted extraction treatment with clear aligners
8. Patients showed good compliance and sticked with their appointments

Inclusion criteria for teeth

1. Maxillary central and lateral incisor teeth were selected as objective teeth for measurement
2. Normal dental crown morphology, no hypoplasia or defects on the enamel, no root canal treatment or history of crown repair

Exclusion criteria

1. Patients with severe periodontitis or temporomandibular joint disease
2. Abnormal crown morphology or root morphology caused by various reasons
3. Patients refused to accept the treatment scheme
4. Poor patients compliance

### Ethical approval

This study was approved by the Ethics Committee of Stomatology Hospital of the Fourth Military Medical University (Reference number: IRB-REV-2021126). All patients provided written and signed informed consent.

### Study group

**Fig. 1:**
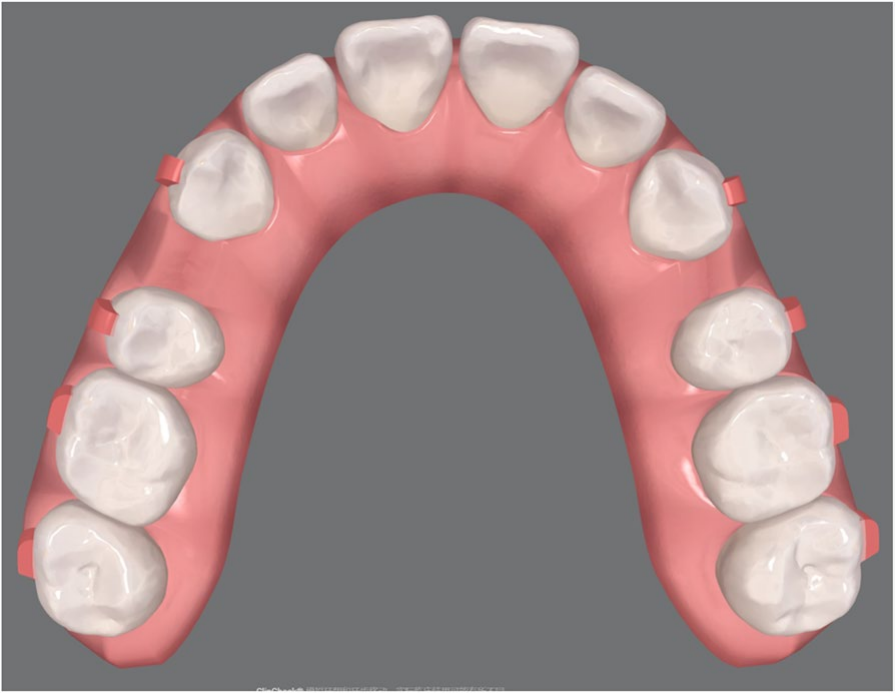
Schematic diagram showing the addition of anterior dental space

In accordance with the inclusion criteria, 24 patients were selected and then randomly assigned to groups using SPSS software. The randomization was as follows: (1) a group with no gaps (9 cases) where the anterior teeth interdental gaps were not increased and the anterior teeth en-mass retraction closed the extraction gap by 2 mm; (2) a group with 0.5 mm gaps (6 cases) where the anterior teeth interdental gaps were increased by 0.5 mm prior to the retraction stage and the anterior teeth en-mass retraction closed the extraction gap by 2 mm; and (3) a group with 1.0 mm gaps (9 cases) where the anterior teeth interdental gaps were increased by 1.0 mm prior to the retraction stage and the anterior teeth en-mass retraction closed the extraction gap by 2 mm (Fig. 1).

**Fig. 2:**
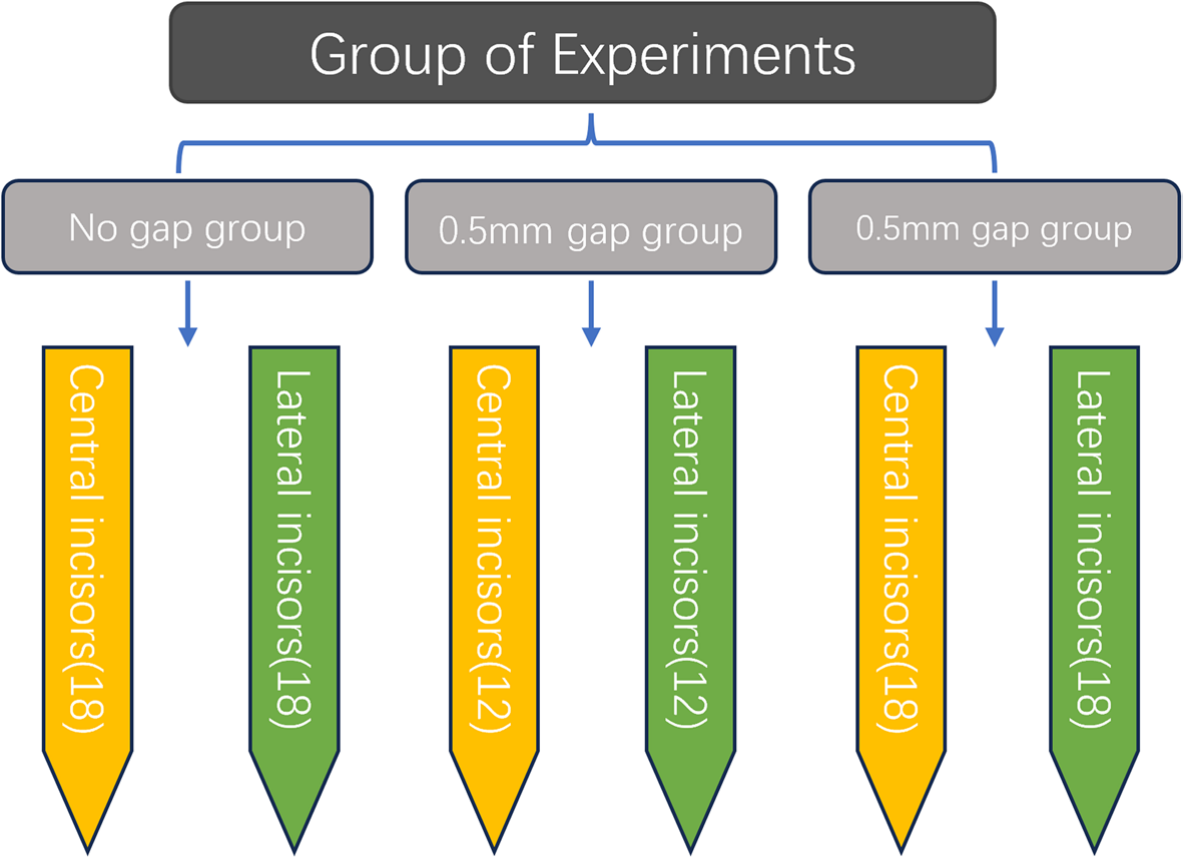
Schematic diagram showing the experimental groupings

According to teeth position, the subjects were divided into a group with central incisors and a group with lateral incisors (Fig. 2). In cases of tooth extraction, clinical observations indicated that the clear aligner technique for en-mass retraction of anterior teeth tended to result in distally oblique positioning of the canines, which led to inadequate control of the canines in terms of three-dimensional movement. In order to address this problem, certain orthodontists choose to employ the approach of moving the canine distally by a distance ranging from one-third to one -half of thew extraction gap before proceeding with overall retraction. When employing this approach, it is possible to increase the distance between the canines and the lateral incisors, as well as the area of crown covered by the appliances. This can enhance our capacity to control the canines. Additionally, this technique is less susceptible to distal crown movement during retraction [7]. Furthermore, due to extensive prior research on canines movement, this study did not include canines as subjects.

### Study design

As a phasic study, we included 10 steps of the retraction stage in our research. The design requirements for the three-dimensional (3D) clinical scheme for clear aligners are described below.

1. The maxillary anterior teeth were distalized sequentially to create an interdental gap. Furthermore, the anterior teeth may undergoing straightening, intrusion, or correction through lip-rooted inclination. At the onset of retraction, a gap of 0/0.5/1.0 mm showed be present between the maxillary teeth (four incisors).
2. Before the en-mass retraction of the six anterior teeth by 2 mm, it is recommended that the anterior teeth not be designed for intrusion or torque movement (crown and root) during the retraction stage. Attachments should not be tailored for incisors, and traction devices, such as bite ramps and hooks, should be avoided. Vertical rectangular attachments ought to be designed for the canines and second premolars. Horizontal rectangular attachments should be designed specifically for molars.
3. There is no specific movement design for the posterior teeth.
4. The designed lip-lingual movement for each step was 0.2 mm. After conducting 10 research steps, we obtained maxillary denture models.
5. After a 2 mm of retraction, we developed a follow-up plan tailored to the individual patient’s specific condition.
6. The patients were instructed to wear the appliance for a minimum of hours per day and to replace the next pair of appliances every 2 weeks.

### Procedure and measurements

**Fig. 3:**
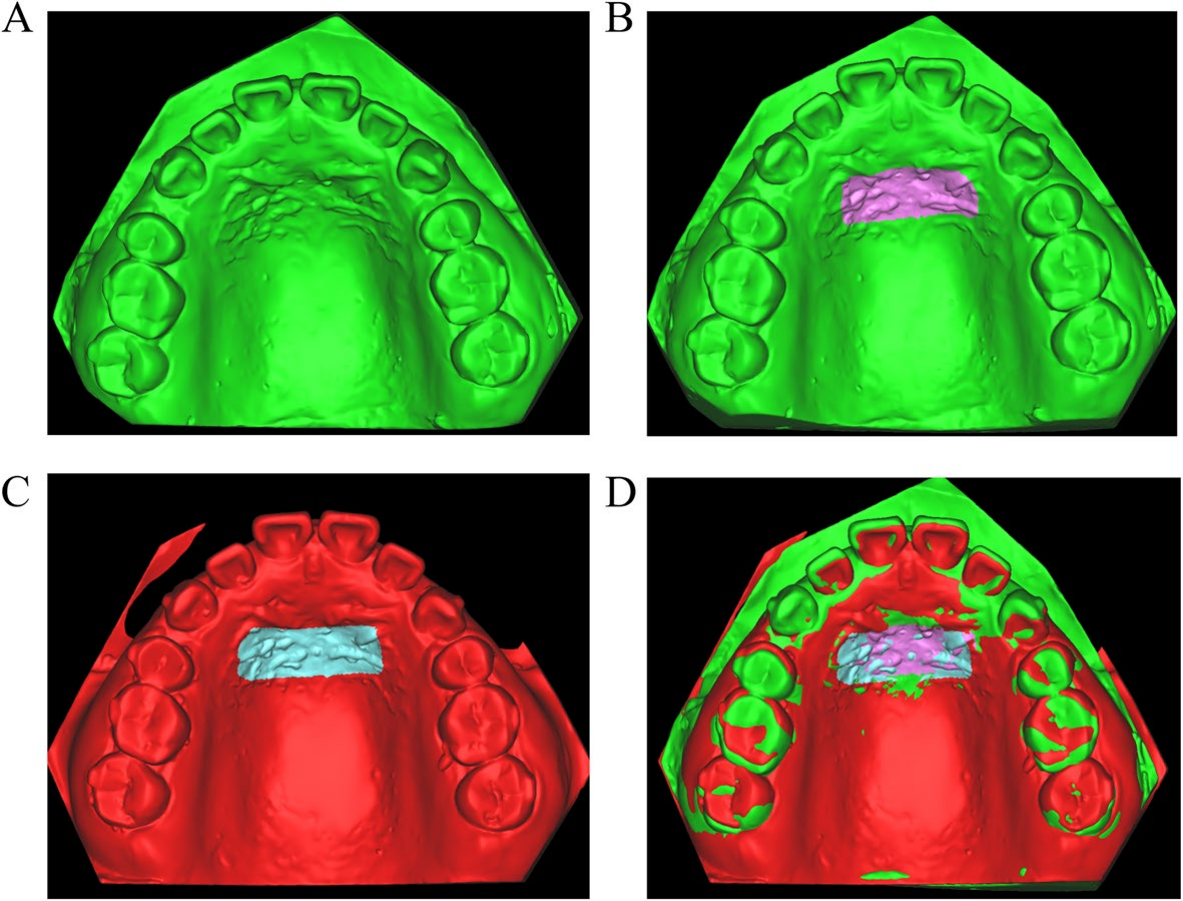
Diagram showing model overlap. **A** Schematic diagram showing maxillary 3D digital model acquisition. **B** Schematic diagram showing the selection of overlapping regions for the T0 model. **C** Schematic diagram showing the selection of overlapping regions for the T1 model. **D** Schematic diagram showing overlap for the maxillary digital model

**Fig. 4:**
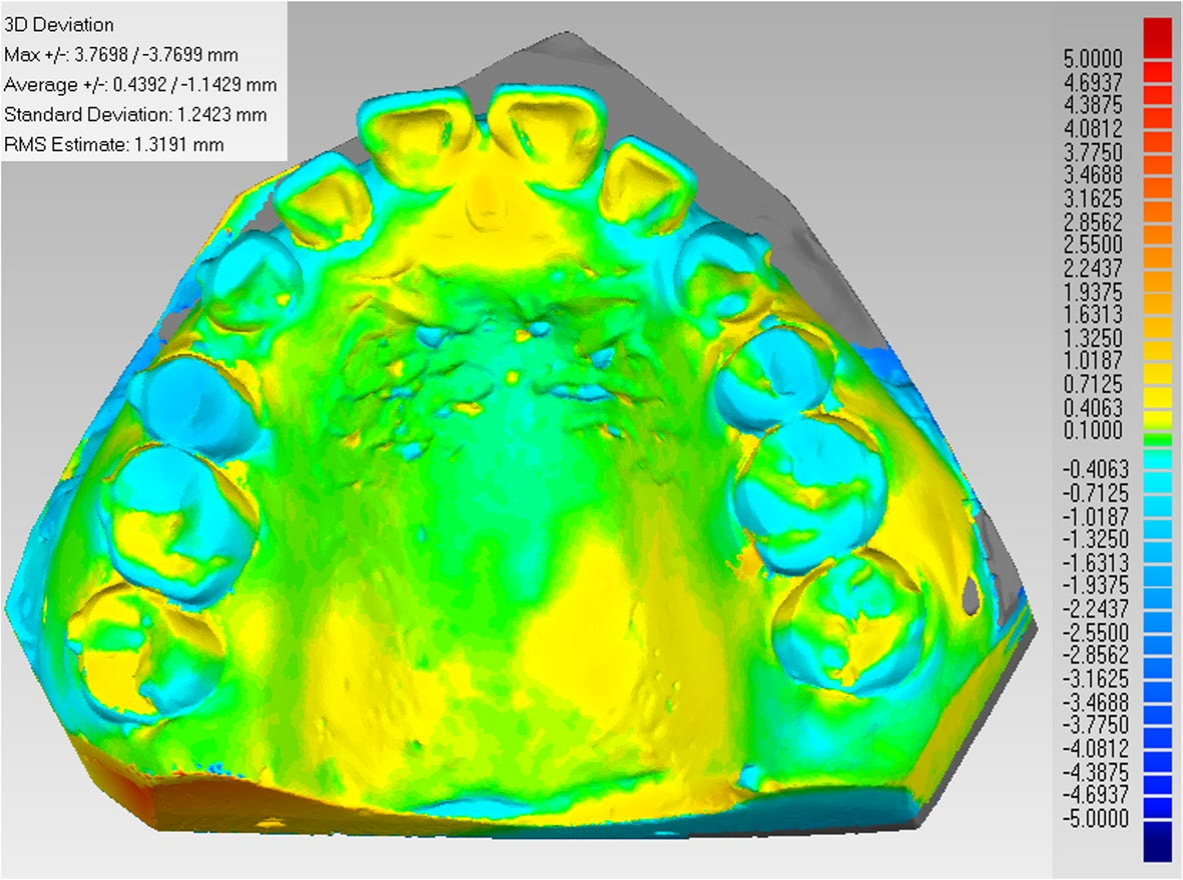
Schematic diagram showing chromatographic deviation analysis for the models

An AR700 laser scanner (3shape, Denmark) was employed for scanning the initial and phased maxillary plaster dental models in order to acquire a 3D digitized model (Fig. 3A), which was subsequently imported into Materialise ProPlan CMF 3.0 software (Materialise Belgium) in STL format. The area encompassing the second and third palatine folds was chosen as the overlapping region. The region encompassing the T0 and T1 models (Fig. 3B and C) was chosen for the purpose of creating a superimposed diagram of the maxillary digital models (Fig. 3D). The precision of model overlap serves as the foundation for accurately measuring experimental data; therefore, it was crucial to verify the reliability of model overlap’s precision. In this study, the deviation chromatographic analysis function in Geomagic Studio 2014 software (3D Systems Company, USA) was employed to assess the degree of alignment between the overlapping maxillary digital models. The analysis of the overlapping area indicated that the morphology of the palatine folds in both models exhibited similarity, and the progression of the overlap demonstrated consistent accuracy (Fig. 4).

**Fig. 5:**
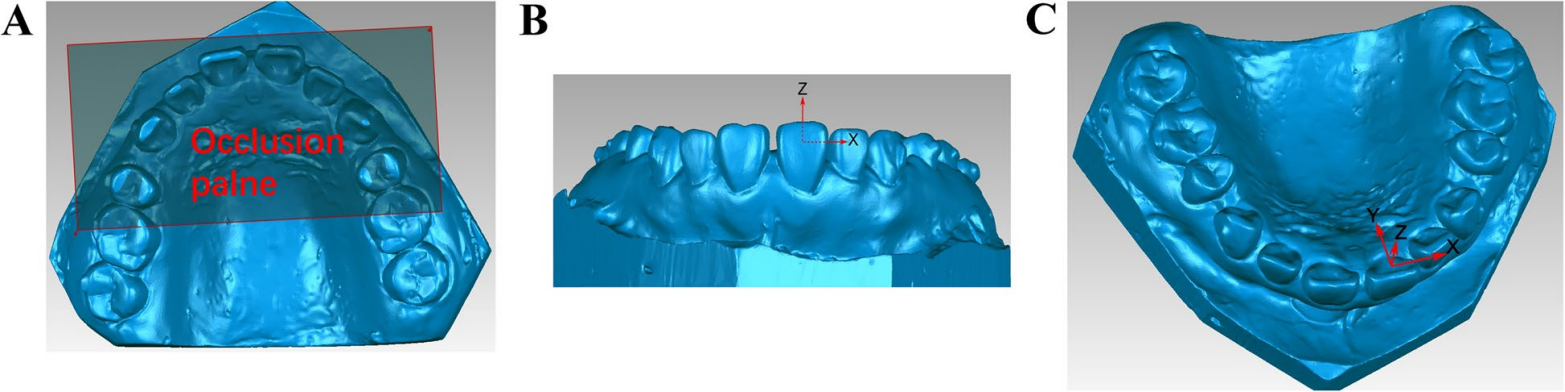
Establishment of congruent planes and coordinate axes. **A** Establishment of the occlusion plane. **B** Establishment of the coordinate system. **C** Schematic diagram showing axis directions for the teeth

To evaluate the movement of teeth, a three-dimensional coordinate system was established for each individual tooth. Firstly, the overlap models were imported into the Geomagic Studio software. Subsequently, an appropriate plane was established, along with the axis, and the direction of the axis was determined. The specific methodology is shown in Fig. 5A-C.

### Torque of the anterior teeth

**Fig. 6:**
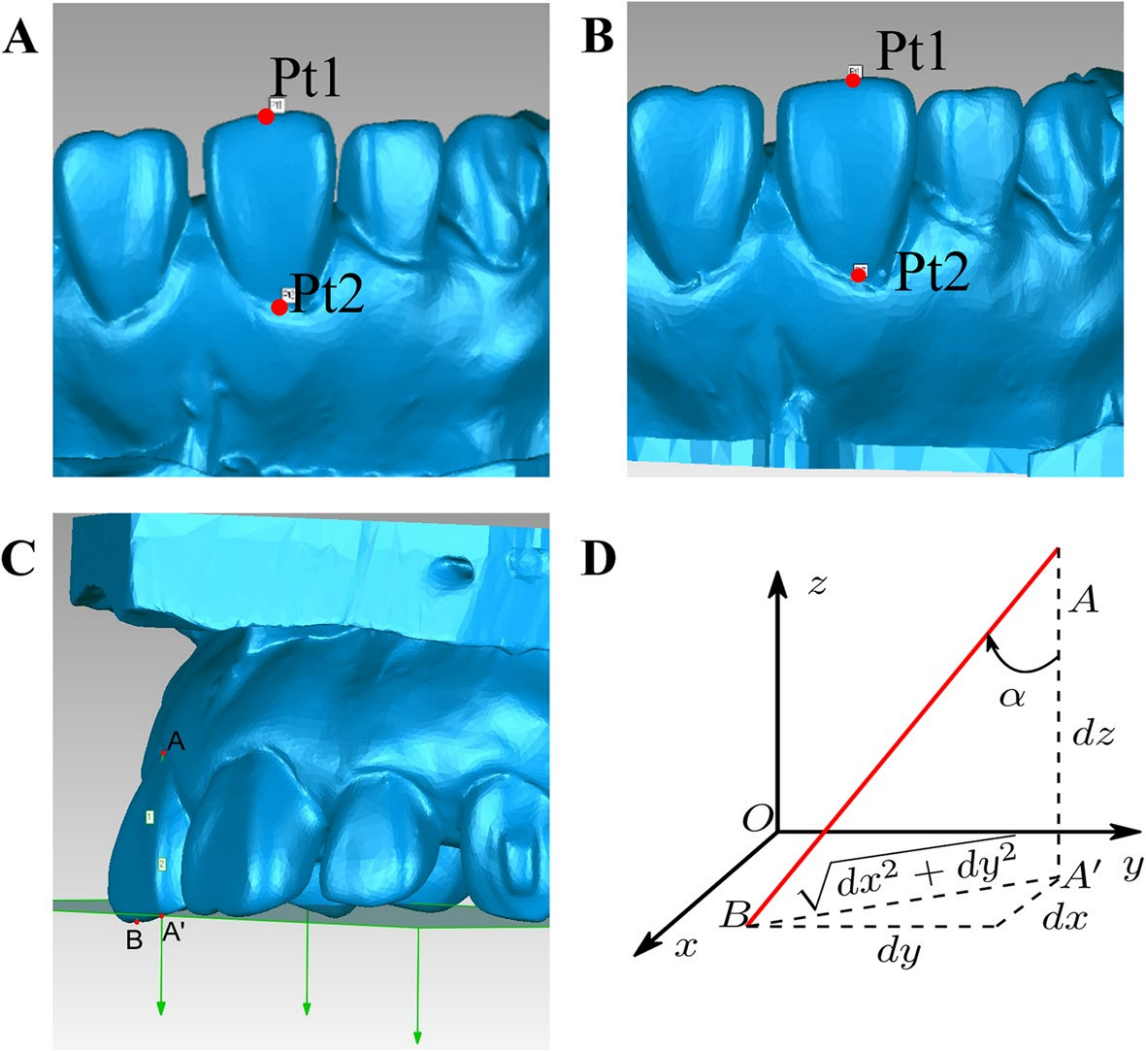
Torque calculation diagram. **A** Selection of marker points for torque measurements in the T0 model. **B** Selection of marker points for torque measurements in the T1 model. **C** Schematic diagram showing the measurement model for torque in the front teeth. **D** Schematic diagram showing the principle used to calculate torque in the front teeth

The midpoint of the incisor margin (pt1) and the most concave point of the labial cervical margin of the central incisors (pt2) were chosen as the measurement markers (Fig. 6A and B).

The crown torque was defined as the angle formed between the clinical crown axis of the tooth and the vertical line of the occlusal plane, which is formed by the midpoint of the incisor and the mesial buccal cusp of the first molars (Fig. 6C). The X-axis represents the mesial-distal movement, the Y-axis represents the labial-lingual movement, and the Z-axis represents the vertical movement of the tooth. Then, we used an inverse trigonometric function was employed to compute the torque of the anterior teeth (Fig. 6D). Point A denoted the most concave point of the labial cervical margin of the tooth, point B represented the midpoint of the incisal margin of the tooth, and point A’ represented the projected point of point A in the XOY plane (Fig. 6D), as follows:

$$\textrm{dx}=\left|{\textrm{x}}_{\textrm{a}}-{\textrm{x}}_{\textrm{b}}\right|, dy=\left|{y}_a-{y}_b\right|, dz=\left|{z}_a-{z}_b\right|,{A}^{\hbox{'}}B=\sqrt{dx^2+{dy}^2}$$

Thus, the torque can be expressed as:

$$\alpha =\arctan \left(\sqrt{dx^2+{dy}^2}/ dz\right)\times 180{}^{\circ}/\pi .$$

Furthermore, the torque change of the anterior teeth before and following retraction was specfied as:

$$\Delta \alpha ={\alpha}_{T1}-{\alpha}_{T0}$$

### Three-dimensional displacement of the anterior teeth

**Fig. 7:**
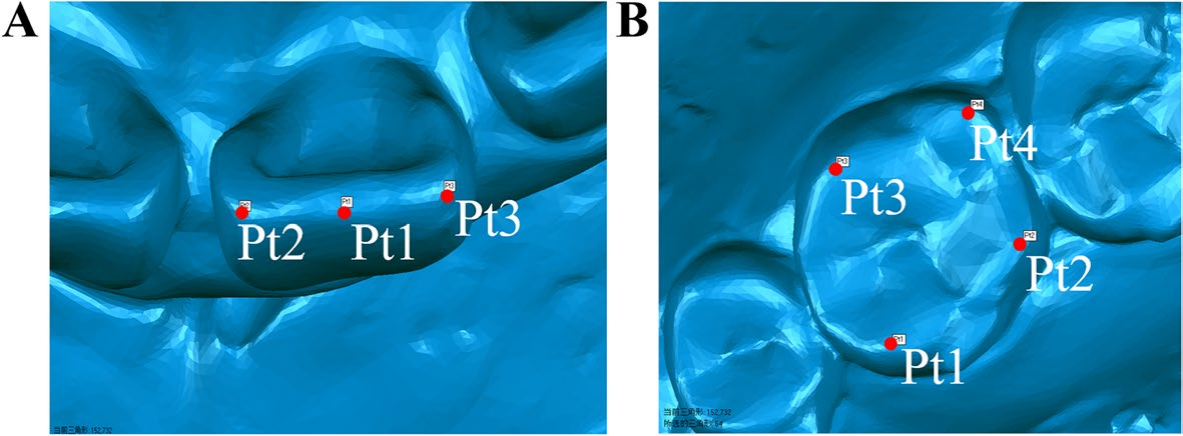
Determination of reference points for tooth movement. **A** The selection of reference points and a measurement data diagram for the anterior teeth. **B** Selection of reference points and a measurement data diagram for the posterior teeth

Displacement in the three-dimensional direction was indicated by the movement of the incisal edge of the anterior teeth. We selected the midpoint of the incisal margin of the maxillary central and lateral incisal teeth (pt1), the intersection of the incisal margin and the mesial marginal ridge (pt2), and the intersection of the incisal margin and the distal marginal ridge (pt3) were chosen as measurement markers (Fig. 7A). The coordinate values of the three reference points were recorded and averaged to represent the positions of the incisal margin of the central and lateral incisal teeth within the three-dimensional coordinate system. The coordinates for the T0 middle and lateral incisors were denoted as (X_0_, Y_0_, Z_0_), and the coordinates for the T1 middle and lateral incisors were denoted as (X_1_, Y_1_, Z_1_).

Next, we measured movement of the anterior maxillary teeth in the labial-lingual (DY), mesial-distal (DX), and vertical (DZ) directions:

$$DY={Y}_1-{Y}_0$$

$$DX={X}_1-{X}_0\;\left(\textrm{in}\ \textrm{zone}\ \textrm{A}\right)$$

$$DX=-\left({X}_1-{X}_0\right)\;\left(\textrm{in}\ \textrm{zone}\ \textrm{B}\right)$$

$$DZ={Z}_1-{Z}_0$$

Using this formula, positive values indicated lingual or distal movement, and negative values indicated labial or mesial movement of the anterior teeth. Positive values denote tooth extrusion and negative values indicate tooth intrusion.

### Three-dimensional displacement of premolars and molars

The following observation points was selected: the buccal cusp (Pt1) and the lingual cusp (Pt2) of the maxillary second premolars, the mesio-buccal cusp (Pt1), the mesio-lingual cusp (Pt2), the distal buccal cusp (Pt3), and the distal lingual cusp (Pt4) of the maxillary first and second molars (Fig. 7B). The mean value of the observation points signifies the variation in displacement within the observed teeth.

Initially, the coordinate values of the observation point in the T0 model were documented in order to calculate the average coordinate values of the first molar, second molar, and the second premolar:

$$\left(X_{\mathit0},\;Y_{\mathit0},\;Z_{\mathit0}\right)$$

For the premolar:

$${X}_0=\left( Pt1x+ Pt2x\right)/2$$

$${Y}_0=\left( Pt1y+ Pt2y\right)/2$$

$${Z}_0=\left( Pt1z+ Pt2z\right)/2$$

For molars:

$${X}_0=\left( Pt1x+ Pt2x+ Pt3x+ Pt4x\right)/4$$

$${Y}_0=\left( Pt1y+ Pt2y+ Pt3y+ Pt4y\right)/4$$

$${Z}_0=\left( Pt1z+ Pt2z+ Pt3z+ Pt4z\right)/4$$

Mean coordinate values were derived from the T1 model using the same methodology as for the T0 model. Subsequently, the movement of the premolars and molars was measured in the labial-lingual (DY), mesial-distal (DX), and vertical (DZ) directions as outlined below:

$$DY={Y}_1-{Y}_0$$

$$DX={X}_1-{X}_0\;\left(\textrm{in}\ \textrm{zone}\ \textrm{A}\right)$$

$$DX=-\left({X}_1-{X}_0\right)\;\left(\textrm{in}\ \textrm{zone}\ \textrm{B}\right)$$

$$DZ={Z}_1-{Z}_0$$

Positive and negative values hold equal significance in representing the three-dimensional displacement of the anterior teeth.

### Sample size calculation

Based on our pre-experimental findings, it was anticipated that the mean movement in the mesial and distal directions for the first, second, and third groups would be 0.71, 0.53 and 0.57, respectively, with a standard deviation of 0.36 (Grasp 1-β = 0.80; test level α = 0.05 (bilateral); group 1: Group 2: Group 3 = 3:2:3). The amount of movement in the mesial and distal direction was calculated using one-way analysis of variance (ANOVA) F-Tests, in accordance with the pre-set parameters. The analysis indicated that the minimum sample size of 216 cases was required for the three groups. Given the 10% rate of loss to follow-up, a total of 240 teeth and 24 patients were enrolled in this study, comprising 9 cases in the first group, 6 cases in the second group, and 9 cases in the third group. These numbers were deemed sufficient to uphold the accuracy and scientific rigor for this study.

### Statistical analysis

Statistical analysis was conducted using the SPSS software (version 27; IBM Corp, Armonk, NY, USA). The data are presented as the mean value plus or minus the standard deviation. A Kolmogorov-Smirnov (K-S) test was conducted to demonstrate the adherence of all data to a normal distribution. The one-way ANOVA was employed to compare between groups, and the SNK-q test was utilized for pairwise comparisons. We established the significance level α at 0.05, and a *P* < 0.05 was deemed to be statistically significant.

## Results

1. Intergroup comparison of central and lateral incisor torque

One-way analysis of variance (ANOVA) was utilized to evaluate the variations in torque changes for the maxillary central and lateral incisors among different groups, as outlined in Table 1. The torque changes for the middle and lateral incisors decreased significantly during the retraction process in the group with additional gaps (*P* < 0.05). Subsequent pairwise comparisons indicated significant differences between the group with a 0.5 mm gap, the group with a 1.0 mm gap, and the group without any gaps. However, there was no statistically significant difference observed between the group with a 0.5 mm gap, and the group with a 1.0 mm gap (*P* > 0.05) (Table 2).

(2) Intergroup comparison of three-dimensional movement in the central and lateral incisors

**Table 1:** Results of torque change of anterior teeth. 1a Intergroup comparison of the torque changes of the central and lateral incisor teeth (x ® + s, °)

Group	central incisor	lateral incisor
No gap group	4.03 ± 0.91°	3.86 ± 0.94°
0.5 mm gap group	1.35 ± 0.97°	1.21 ± 0.89°
1.0 mm gap group	1.74 ± 0.86°	1.75 ± 0.95°
F	41.69	36.294
P	0.000	0.000

**Table 2:** SNK-q test of the torques of the central and lateral incisors

Group		central incisor		lateral incisor	
	N	Alpha = 0.05		Alpha = 0.05	
		1	2	1	2
No gap group	18	4.029		3.864	
0.5 mm gap group	12		1.738		1.208
1.0 mm gap group	18		1.348		1.750
sig		1.000	0.238	1.000	0.114

One-way ANOVA was employed to assess the differences in the three-dimensional displacement of the central and lateral incisors between groups (Table 3). During the process of retraction, changes in the labial-lingual and vertical directions for the middle and lateral incisors decreased significantly in the groups where gaps were added (*P* < 0.05). There was no statistical significance in terms of displacements between the central and lateral incisors in the mesial and distal directions (*P* > 0.05). The statistically significant data were subsequently analyzed using the SNK-q test (Tables 4 and 5). The labial-lingual and vertical displacements of the central and lateral incisors differed significantly between the groups with additional gaps and the groups without gaps. There was no statistically significant difference observed between the group with a 0.5 mm gaps and the group with a 1.0 mm gaps (*P* > 0.05).

(3) Intergroup comparison of three-dimensional movement in molars

**Table 3:** Results of three dimensional displacement of anterior teeth. 2a Incisor three-dimensional direction displacement change is compared between group (x ® + s, mm)

Location	Direction	No gap group	0.5 mm gap group	1.0 mm gap group	F	P
central incisor	Proximal and distal	0.69 ± 0.40	0.55 ± 0.39	0.56 ± 0.33	0.696	0.504
	Lip-lingual orientation	2.34 ± 0.40	1.61 ± 0.27	1.69 ± 0.44	17.186	0.000
	vertical	0.78 ± 0.51	0.46 ± 0.24	0.25 ± 0.47	6.494	0.003
lateral incisor	Proximal and distal	0.72 ± 0.34	0.65 ± 0.29	0.62 ± 0.36	0.457	0.636
	Lip-lingual orientation	1.98 ± 0.30	1.49 ± 0.43	1.58 ± 0.28	10.183	0.000
	vertical	0.90 ± 0.56	0.43 ± 0.36	0.19 ± 0.64	7.515	0.002

**Table 4:** SNK-q test of three-dimensional displacement changes of incisor teeth

		Proximal and distal		Lip-lingual orientation		vertical	
Group	N	Alpha = 0.05		Alpha = 0.05		Alpha = 0.05	
		1	2	1	2	1	2
No gap group	18	0.686			2.339		0.780
0.5 mm gap group	12	0.547		1.611		0.465	0.465
1.0 mm gap group	18	0.563		1.692		0.251	
sig		0.554		0.567	1.000	0.186	0.054

**Table 5:** SNK-q test of three-dimensional displacement changes of lateral teeth

		Proximal and distal		Lip-lingual orientation		vertical	
group	N	Alpha = 0.05		Alpha = 0.05		Alpha = 0.05	
		1	2	1	2	1	2
No gap group	18	0.772			1.977		0.900
0.5 mm gap group	12	0.648		1.489		0.433	
1.0 mm gap group	18	0.618		1.577		0.191	
sig		0.665		0.457	1.000	0.233	1.000

Finally, a one-way analysis of variance (ANOVA) was employed to compare the alterations in displacement in the three-dimensional direction of the anchorage molars among the three groups (Table 6). There were no statistically significant differences observed in the displacement changes of the second premolars, first molars, and second molars in the bucco-lingual or vertical directions when comparing across the three groups (*P* > 0.05). Significant differences were observed in the mesial and distal displacements of the second premolar, the first molar, and the second molar when comparing the three groups (*P* < 0.05). There were significant differences in the mesial and distal displacements of the second premolar among the three groups. The mesial and distal displacements of the first molar and the second molar exhibited significantly greater values in the groups with additional gaps. Nevertheless, no significant disparity was observed between the group with a 0.5 mm gap and the group with a 1.0 mm gap (Table 7).

**Table 6:** Results of three dimensional displacement of posterior teeth. 1a Posterior teeth three- dimensional direction displacement change is compared between group (x ® + s, mm)

Location	Direction	No gap group	0.5 mm gap group	1.0 mm gap group	F	P
Second premolar	Proximal and distal	−0.77 ± 0.35	−1.17 ± 0.39	−1.49 ± 0.37	17.209	0.000
	Buccal-lingual orientation	− 0.51 ± 0.36	− 0.61 ± 0.63	− 0.51 ± 0.54	0.17	0.884
	vertical	−0.38 ± 0.38	− 0.50 ± 0.33	− 0.62 ± 0.56	1.269	0.291
First molar	Proximal and distal	−0.46 ± 0.29	−0.86 ± 0.23	− 0.91 ± 0.40	10.325	0.000
	Buccal-lingual orientation	−0.31 ± 1.24	−0.50 ± 0.82	− 0.87 ± 0.52	1.706	0.193
	vertical	−0.55 ± 0.26	−0.85 ± 0.75	− 0.91 ± 0.44	2.754	0.074
Second molar	Proximal and distal	−0.49 ± 0.31	−0.81 ± 0.32	− 0.96 ± 0.39	8.846	0.001
	Buccal-lingual orientation	−0.07 ± 1.48	−0.46 ± 0.47	− 0.36 ± 0.84	1.119	0.336
	vertical	−0.65 ± 0.35	−0.62 ± 0.31	− 0.71 ± 0.69	0.126	0.882

**Table 7:** SNK-q test for comparison of displacement changes of anchorage molars in the proximal and distal directions between groups

		Second premolar			First molar		Second molar	
Group	N	Alpha = 0.05			Alpha = 0.05		Alpha = 0.05	
		1	2	3	1	2	1	2
No gap group	18	−0.771			−0.456		− 0.489	
0.5 mm gap group	12		−1.175			−0.863		−0.812
1.0 mm gap group	18			−1.490		−0.908		−0.962
sig		1.000	1.000	1.000	1.000	0.699	1.000	0.230

## Discussion

Prior to obtaining the model, the patients were instructed to thoroughly clean their oral cavity. Then, we verified that the morphology of the palatal rugae in each participant corresponded to that of the plaster model. Any discrepancies or imperfections were identified and rectified to guarantee the authenticity and precision of the research model, with particular attention to the palatal rugae and tooth surfaces [8]. If necessary, we obtained the research model again. Recent evidence indicates that alterations in the anatomical positioning of the rugae palatine remain relatively stable throughout craniofacial growth, development, and orthodontic treatment, and they are commonly considered as reference points for the alignment of three-dimensional images [9, 10]. In cases involving extraction, it is important to note that the position of the maxillary first rugae palatine is unstable and therefore should not be relied upon as a reliable landmark. Therefore, the area encompassing the second and third palatine folds was chosen as the overlapping region in this study. The accuracy of the 3D invisible appliance may be affected by the precision of the prototyping technology, leading to variations in the preset gap among different 3D printing technologies/systems [11]. Antonino Lo Giudice et al. [12] stated that consumer-grade LCD-based 3D printers at the entry level exhibit lower accuracy compared to professional-grade 3D printers, yet their precision remains comparable to the clinically accepted threshold values in orthodontics.

The main concern and obstacle in the use of clear aligners for treatment are mainly related to the control of tooth movement, particularly in cases involving tooth extraction. The overall stiffness of clear aligners is comparable to that of the nickel-titanium round wire used in fixed appliances, enabling effective tooth alignment. However, achieving a tooth movement effect similar to that of a stainless-steel wire in fixed appliances is challenging when closing the tooth extraction space, primarily due to the long-distance deformation of the appliance and the limited control of the teeth by the edge of the appliance’s edge. The lack of precise control over the three-dimensional movement of the teeth leads to greater movement of the crown compared to the root of the anterior teeth. This results in oblique tooth movement, loss of torque, and increased susceptibility to lingual inclination, extrusion, and deepening of the overbite of the anterior teeth [13].

At present, it is a prevailing belief that clear aligners have limited capacity to control the root movement during orthodontic treatment [14]. Prior research has identified a notable disparity between the predetermined torque and the actual torque expression value in clear aligner treatment [15]. In a prior investigation, Hahn et al. found that the use of clear aligners for correction result in labial-palatine-oriented force as well as side forces along the long axis of the tooth. These side forces can exert a depressing force on the root of the tooth, thereby hindering the establishment of effective coupling achieve a predetermined root movement control [16]. In order to enhance the efficiency of torque expression, a “torque ridge” was incorporated on the cervical incisor to facilitate effective coupling. Furthermore, the torque of the anterior teeth was regulated to facilitate comprehensive tooth movement. Simon et al. found that in cases of tooth extraction with a preset torque exceeding 10°, there was an approximate 50% reduction in torque during the retraction phase. This phenomenon occurred regardless of whether the attachment or the torque ridge was added to the orthodontic device [17]. Prior research has also demonstrated that the application of integral retraction to a maxillary incisor by 0.15 mm resulted in a tendency for distolingual inclination of the tooth. The addition of 5° of lingual torque resulted in an increase in the equivalent stress on the periodontal membrane. Additionally, the trend for the inclination movement of the maxillary incisors approached whole tooth movement, although it was not entirely aligned with it [18]. Therefore, t additional clinical studies are required to validate the efficacy of this approach in enhancing torque on the anterior tooth. In a separate case report on clear aligners and reduction therapy, Meng et al. [19] reported that when retracting the anterior teeth, they designed a 0.5–1.0 mm loose gap between the anterior teeth. This design increased the coverage area of the aligners on the anterior teeth, improved control of the anterior tooth axis, and resulted in a favorable corrective outcome. However, no systematic experimental study has further investigated this effect. In a separate investigation, Tepediion et al. [6] recruited 39 patients with dental crowding < 6 mm and implemented interproximal enamel reduction (IPR) to create space. After wearing 12 sets of clear aligners, the researchers assessed the alterations in the torque of the patients’ anterior teeth. The authors found that there was no statistically significant difference between the torque variation specified in the correction plan and the actual value.

Therefore, increasing the gap between the adjacent teeth using IPR in clear aligners could enhance the torque expression and minimize torque loss. Through the application of three-dimensional limited analysis, Hu et al. [20] found that the inclination movement of the anterior teeth gradually decreased as the anterior tooth space increased during the retraction of anterior teeth. This phenomenon could be explained by the expansion of the anterior interdental space through sequential distant displacement before initiating retraction, as well as the enlargement of the mesial and distal coverage of the incisor tooth by the orthodontic device. These adjustments contribute to improved control over three-dimensional movement of the tooth. Therefore, the alterations in torque for the central and lateral incisors were marginally reduced in the cohort with expanded anterior space. This suggests that augmenting the anterior space facilitated the regulation of anterior torque, leading to a greater inclination towards overall movement in the anterior teeth. The results were in line with the three-dimensional finite element analysis that had been previously documented by Hu et al [20] Therefore, in clinical practice, enhancing our control of torque in relation to the anterior teeth can be achieved by expanding the anterior tooth space while simultaneously closing the extraction space. This approach has the potential to minimize the loss of torque during the retraction process.

Accurately controlling the three-dimensional movement of the anterior teeth is essential for successfully correcting extraction cases using clear aligners. The clear aligner appliance applies a therapeutic force by encasing the tooth crown and the modifying the appliance. The natural chewing force induces the “jaw cushion effect”, causing posterior tooth intrusion [21]. Furthermore, the reaction force from the posterior tooth intrusion leads to extrusion of the anterior teeth, resulting in increasing overbite and premature contact of the anterior teeth during the treatment process. However, modifying the increasing overlap during the later stage can be a time-consuming and laborious process. The solution to addressing a deepening overbite involves the intrusion of anterior teeth. The decrease in tooth extraction results in the formation of a cavitation structure within the space where the tooth was extracted; which disrupts the transmission of orthodontic force and diminishes the transmission of force for the anterior tooth intrusion [22]. Patients with bimaxillary protrusion may experience a more pronounced “bow effect” when using clear aligner devices, as noted in a study by [23]. This effect can lead to a “roller coaster effect,” which in turn complicates the management of vertical tooth positioning, as discussed in a study by [24]. Gu et al. found that there was a specific correlation between the retraction design and the efficiency of the anterior teeth intrusion. When the anterior teeth were not intended for retraction, the efficiency of the anterior tooth intrusion movement was 46.9%. however, when the retraction was incorporated into the design, the anterior tooth intrusion movement was not achieved; instead, tooth extrusion was observed. In another study, Song et al. [25] observed the absence of intrusion but the presence of extrusion when a 0.1 mm vertical intrusion displacement was designed for each step during anterior teeth retraction in cases involving tooth extraction (Table 2). Consequently, in the context of employing a clear aligner for extraction cases, it is crucial to augment the intrusion of the anterior teeth while undergoing the retraction process to mitigate the exacerbation of the overlap. However, the comprehensive treatment outcome frequently proves to be unsatisfactory. Ting et al. [26] conducted a three-dimensional finite element analysis and observed that a 0.2 mm retraction of an anterior tooth with a 0.15 mm intrusion per step resulted in a tendency for lingual movement of the tooth root. This movement contributed to the prevention of lingual inclination of the anterior tooth. However, the actual clinical impact of this phenomenon has not been investigated.

There was no planned anterior tooth intrusion for the three groups during retraction. Subsequent to conducting overlapping analysis, it was observed that the vertical alterations in the three groups of anterior teeth were elongated, which align with the results documented by Song et al. [25] in prior studies. However, it was observed that the vertical extrusion of the middle and lateral incisors in the groups with a 0.5 mm/1.0 mm gap compared to the group without gap. The observed variation in extrusion was determined to be statistically significant (Table 3). These findings showed that utilizing a clear aligner to widen the space between the anterior teeth had more favorable impact on vertical alignment. Tooth intrusions must occupy the space with the dental arch. In clinical practice, it is common to retract the anterior teeth without creating additional space between them. However, this approach does not accommodate tooth intrusions, resulting in a suboptimal aesthetic outcome for tooth intrusions. By expanding the anterior tooth space, the orthodontic device’s tooth wrapping area was increased, leading to enhanced control of tooth torque. This expansion also facilitated downward tooth movement and minimized tooth extrusion during the retraction stage. By implementing this strategy, dentists may enhance the efficiency tooth intrusion, minimize torque loss in the anterior teeth during the adduction process, and mitigate the excessive overlap resulting from inefficient expression of intrusion movement.

The analysis revealed that the anterior retraction expression effect was significantly lower in the groups with added gaps compared to the group without gap (Table 3). This phenomenon may be attributed to the expansion of the anterior interdental space, which results in a larger area covered by the orthodontic device. This expansion enhances the practitioner’s capacity to control the movement of the teeth, thereby aligning the mode of anterior teeth movement more closely with the overall movement pattern. Therefore, the displacement of the anterior teeth in the sagittal direction was smaller in the group without gap compared to the amount achieved in the other group. These findings indicate that adjusting the retraction amount for each step may be beneficial in reducing the treatment duration. Yuan et al. [27] performed a three-dimensional finite element analysis to investigate the effect of increasing anterior dental space in clear aligner. They observed that an increase in the anterior dental space led to a more pronounced tendency for the anterior teeth to move as a body rather than inclined to move, resulting in decreased periodontal membrane stress. This finding aligns with the results of our own experimental study.

Due to a lack of tooth control, we often insufficient torque control of the anterior teeth during the retraction stage with clear aligners. Furthermore, the anterior tooth intrusion efficiency is often low; this results in a “roller coaster” effect and a poor fit between the appliance and the teeth. This leads to an off-track effect which requires redesign or re-manufacture of the appliance and elongates the period of clinical treatment. In this study, despite the sequential remote displacement leading to increase the anterior dental space would increase the number of correction steps, this method had better control over anterior tooth torque and vertical direction. This made the anterior teeth less prone to torque loss and contributed to an increased deepening of the overlap during the retraction process; this also prolonged the period of treatment.

In the present study, it was observed that mesial, buccal inclination, and extrusion movement were evident in all groups, which aligns with the findings reported by Gu et al. [28]. Inter-group comparisons were conducted to analyze the changes in the three-dimensional direction of the three groups of anchorage molars. The results indicated that the inclined buccal movement and vertical extrusion of the anchorage molars in the groups with added gap were greater than those in group without gap. However, these differences were not found to be statistically significant (Tables 6, 7). The mesial movement of the anchorage molars in the groups with added gap was significantly higher compared to the group without gap (Tables 6, 7). However, there was no statistically significant difference in the mesial movement when comparing the groups with a 0.5 mm and 1.0 mm gap added.

Based on our analysis, it was found that augmenting the anterior space resulted in a more effective control of torque on the anterior teeth. Furthermore, the anterior teeth exhibited a mode of movement that was more similar to the overall movement. The principle of orthodontic biomechanics indicates that as the anterior retraction movement aims to model the entire process, there is a greater need for anchorage. Therefore, the loss of molar anchorage was higher in the groups where a gap was added compared to those in the group without gap. Notably, no methods were employed to enhance anchorage during the retraction stage. Consequently, it is necessary to incorporate additional anchorage-enhancing designs in the initial phases of treatment. Thus, increasing the anterior tooth space would not result in additional consumption of throughout the treatment process, and it would also prevent any extension of the treatment period.

## Conclusion

As a prospective study, our investigation was grounded in the phased digital overlap model, and we established three experimental groups using clear aligners for extraction cases. By increasing the anterior tooth space during the retraction stage and closing the extraction space by 2 mm, alterations in torque, the three-dimensional displacement of the anterior teeth, and the three-dimensional displacement of the anchorage molar teeth were observed. Based on our analysis, the following conclusions can be drawn: (1) increasing the adduction mode of the anterior tooth space facilitated the regulation of torque in the anterior teeth and resulted in a movement mode more inclined to overall movement; (2) increasing the adduction mode of the anterior tooth space may decrease the extrusion of the anterior teeth and help to control the vertical direction of the anterior teeth; and (3) increasing the adduction mode of the anterior space will lead to an increased loss of molar anchorage. Hence, it is essential to focus on anchorage control during the clinical design period. However, with regards to the entire correction process, this method does not result in increased loss of anchorage.

## Data Availability

All relevant datasets and their supporting information files generated and/or analyzed during this study are available from the corresponding author upon reasonable request.

## Abbreviations

3D: Three-dimensional
IPR: Interproximal enamel reduction
